# Factors affecting neurodevelopmental outcome following surgical necrotising enterocolitis: a systematic review

**DOI:** 10.1007/s00383-024-05651-x

**Published:** 2024-03-06

**Authors:** E. I. Okten, M. Frankl, S. Wu, H. Gamaty, H. Thompson, I. E. Yardley

**Affiliations:** 1 GKT School of Medical Education, King’s College London, London, UK; 2 Department of Paediatric Surgery, Evelina London Children’s Hospital, London, UK

**Keywords:** Necrotising enterocolitis, surgery, Neonatal surgery, Infant, Neurodevelopmental outcomes, impairment

## Abstract

Surgically treated necrotising enterocolitis (sNEC) is associated with significantly worse neurodevelopmental outcomes than that seen in premature infants without NEC. We aim to review the association between factors involved in the surgical treatment of NEC and subsequent neurodevelopmental outcomes to identify potential areas for improvement. The PubMed and Embase databases were interrogated for articles reporting neurodevelopmental outcomes in babies treated surgically for NEC using key terms including: “Infant”, “Necrotising enterocolitis”, “Surgical”, “Neurodevelopmental” and “Outcomes”. The search strategy yielded 1170 articles and after applying inclusion and exclusion criteria 22 studies remained and formed the review. A diverse range of neurodevelopmental outcomes were reported. Extreme prematurity and lower birth weight were associated with worse neurodevelopmental outcomes. The use of peritoneal drains and enterostomies were associated with worse outcomes. Modifications to surgical strategies in NEC may improve neurodevelopmental outcomes but the effect of confounding factors remains unclear. Further large scale studies are required to define the optimum strategies for treating NEC surgically and to develop a core outcome set for research into NEC.

**Supplementary Information:**

The online version contains supplementary material available at 10.1007/s00383-024-05651-x.

## Introduction

Necrotising enterocolitis (NEC) is an inflammatory gastrointestinal disease arising in neonates; it is one of the most common causes of mortality and morbidity in babies born prematurely [1]. The UK incidence of NEC in premature infants is 27.9 cases per 100,000 live births, with an overall mortality rate of 23.5% [2]. One in four babies with NEC will undergo surgical intervention, with mortality rates as high as 50.9% in infants with birthweights under 1000 g [3, 4]. It is known that outcomes, including neurodevelopmental outcomes, are worse in infants with NEC than those without, and even worse in those treated surgically compared to those whose NEC is managed non-operatively [5, 6]. Neurodisability following surgery for NEC occurs more frequently than intestinal failure, in as many as 60% of cases [4]. Despite this association between surgical NEC (sNEC) and worse neurodevelopmental outcomes being clearly established, there is limited understanding of the factors that contribute to these worse outcomes. These factors may be non-modifiable and related to the babies’ intrinsic condition or status, or they may be potentially modifiable, for example timing of surgery, and offer potential to improve outcomes in sNEC cohorts [7–9]. Previous reviews have examined outcomes following NEC overall, but a gap remains in reviewing the impact of sNEC on neurodevelopment [4].

Hence, the aims of our study were to:

- Identify studies reporting neurodevelopmental outcomes in sNEC infants;
- Investigate which factors in the management of sNEC are associated with neurodevelopmental outcomes and are potentially modifiable, so representing areas where changes in management could lead to improved outcomes in future sNEC cohorts.

## Methods

### Protocol and registration

A systematic review protocol was registered with PROSPERO [Code: CRD42022370309] on 31/10/2022.

### Data sources and search strategy

The PubMed and Embase databases were interrogated on 01/11/2022, using key terms including: “Infant”, “Necrotising enterocolitis”, “Surgical”, “Neurodevelopmental”, and “Outcomes”. The full search strategy can be found in Appendix 1. The inclusion and exclusion criteria are as listed in Table 1, publication after the year 2000 was used in an attempt to ensure that included studies were relevant to current practice.

**Table 1:** Inclusion and exclusion criteria

Inclusion criteria
Reports patients undergoing surgical intervention for NEC
Reports neurodevelopmental outcome as a discrete measure (i.e., not compounded with another outcome such as death)
Human studies
Published after 2000
Cohort studies, comparative studies, randomised control trials
Full text available in English language
Exclusion criteria
Case reports, case studies or literature reviews

### Study selection

Records identified by the search strategy were collated and deduplicated. The resulting articles were screened by title and abstract against the inclusion/exclusion criteria. The remaining articles underwent full-text review by at least two authors, and any disagreements on eligibility for inclusion were resolved by discussion and consensus. Although review articles were excluded, the references were hand searched for any papers meeting our inclusion criteria.

### Quality of included studies

The Quality in Prognosis Studies (QuIPS) tool was used to assess the quality of studies included [10]. Six domains were assessed: (1) study participation, (2) study attrition, (3) prognostic factor measurement, (4) outcome measurement, (5) study confounding, and (6) statistical analysis and reporting. For all papers, each domain was assessed as either: “sufficiently reported”, “moderately reported”, or “not reported”. Based on this, each paper and domain were graded overall as being either: “low risk”, “medium risk”, or “high risk”, in terms of potential bias.

## Results

### Study identification

**Fig. 1:**
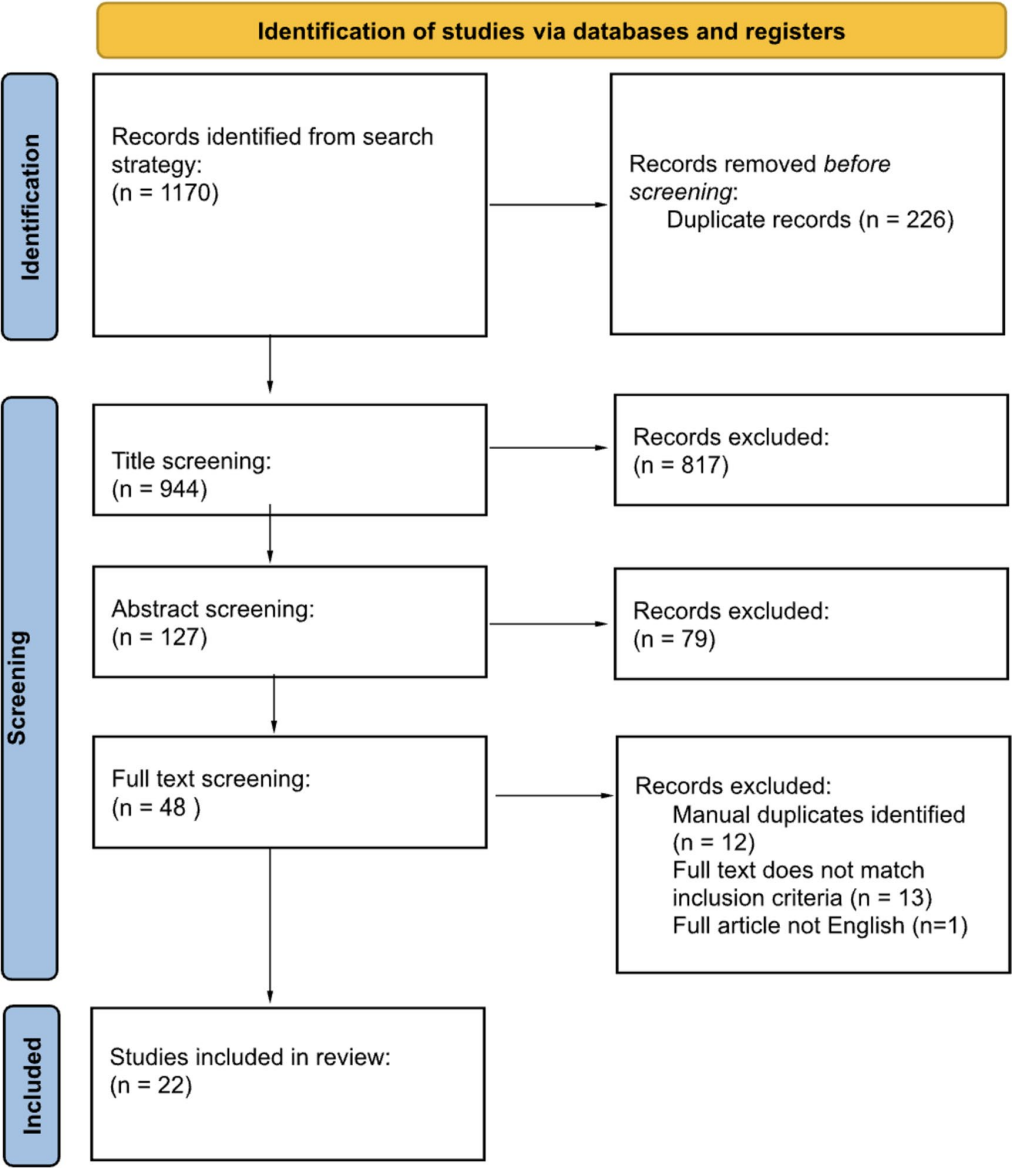
Prisma flowchart

The initial search strategy yielded 1170 articles. After deduplication and screening against inclusion and exclusion criteria, 22 remained to form the review (Fig. 1).

### Study findings

Of the 22 studies included, there were 15 retrospective, 6 prospective, and 1 prognosis study. The study characteristics are shown in Table 2, organised by study type (prospective first), and cohort size. We have not reported in detail the outcomes presented in the included studies as this fell outside the primary aim of our review, to identify factors associated with neurodevelopmental outcomes following sNEC.

**Table 2:** Characteristics of included studies

Author	Study characteristics			NEC patient characteristics				
	Study type	Neurodevelopmental outcome reported	Risk of Bias	Total number of participants	Number of participants with surgical NEC	Mean birth weight (g),	Mean gestational age (weeks)	Duration of follow-up
Fullerton et al., (2018) [8]	Prospective cohort study	Bayley MDI or PDI, bilateral blindness, hearing impairment, cerebral palsy	Low	9063	449	Medical NEC 799 ± 162 Surgical NEC 752 ± 172	Medical NEC 26 ± 2 Surgical NEC 25 ± 2	18–24 months
Blakely et al., (2021) [28]	Prospective multicenter observational	Bayley MDI and PDI	Medium	992	310	Drain 711.4 Laparotomy 721.2	Drain 24.9 Laparotomy 25.1	18–22 months
Humberg et al., (2020) [15]	Prospective multicenter cohort study	IQ scores, motor function	Low	2241	43	810 ± 268	NEC 26.3	6 years
Vaidya et al., (2022) [24]	Prospective longitudinal study	DAS-II, LPA, IQ, Wechsler abbreviated scale of intelligence-II, ADHD assessment Cerebral palsy,	Low	1506	33	N/A	23–27	10 and 15 years
Merhar et al., (2014) [9]	Prospective study	Level of brain damage from Brain Magnetic Resonance Imaging	Medium	29	6	All infants 925 Medical NEC 1150 Surgical NEC/SIP 790	All infants 27.4 Medical NEC 27.4 Surgical NEC 26.1	40 weeks
Shah TA et al., (2011) [18]	Prospective cohort	WISC-III-NLM-ABC, MDI, and PDI,	Medium	1722	121	Medical NEC 769 Surgical NEC 753	Medical NEC 26.1 Surgical NEC 25.7	18–22 months
Ayed et al., (2014) [27]	Retrospective multicenter study	Intraventricular haemorrhage, periventricular (IVH) leukomalacia (PVL)	Medium	11,974	214	Medical NEC 1113 Surgical NEC 941	Medical NEC 27.5 Surgical NEC 26.4	36 weeks
Wadhawan et al., (2013) [14]	Retrospective cohort study	Bayley PDI and MDI Amiel-Tison assessment, cerebral palsy, hearing and visual impairment	Low	9507	472	Surgical NEC 736	Surgical NEC 25.6	18–22 months
Hintz, (2005) [13]	Retrospective multicenter study	BSID-II, diagnosis of cystic periventricular leukomalacia, MDI, and PDI	Low	2948	124	401–1000	< 28	18–22 months
Ganapathy et al., (2013) [12]	Retrospective study	Neurodevelopmental delay, failure to thrive, cerebral palsy	High	3159	73	N/A	N/A	6–12 months
Adesanya et al., (2005) [16]	Retrospective observational cohort study	Bayley score-II, PDI and MDI	Medium	1357	39	< 1500, NEC 841 ± 212	26 ± 2	12 months
Martin et al., (2010) [30]	Retrospective cohort study	BSID-II, Gross Motor Functional Classification System, developmental dysfunction and microcephaly association	Medium	1155	42	N/A	23–27	24 months
Shah et al., (2011) [18]	Retrospective cohort study	BSID-II, incidence of IVH, PDA, PVL	Medium	865	32	Surgical NEC 753 Medical NEC 769	Medical NEC 26.1 Surgical NEC 25.7	18–22 months
Han et al., (2020) [29]	Prognosis study	Neurodevelopmental disability	Medium	268	268	930	28	8 years
Garg et al., (2021) [6]	Retrospective study	Bayley’s BSID-III, MDI, PDI, WBMI on MRI scans	Medium	243	121	927 ± 484	26.5 ± 2.7	24 months
Arnold et al., (2010) [19]	Retrospective review	Neurodevelopmental delay, auditory deficit, visual impairment	Medium	128	119	1413	32	39 months
Roze et al., (2011) [20]	Retrospective case–control study	WISC-III-NL, IQ level, cerebral palsy, motor and behavioural impairment	Low	93	17	Medical NEC 1100 Surgical NEC 1411 SIP 1173 control 1220	28.9	6–13 years
Shin et al., 2021) [31]	Retrospective single centre study	Bayley-III, K-ASQ, K-DST Gross motor, fine motor, cognitive and social domains	Low	82	60	Surgical NEC 710	Surgical NEC 26.6	24 and 36 months
Cuzzilla et al., (2014) [11]	Retrospective chart audit	Bayley score-III and ASQ	High	45	45	Surgical NEC < 1500	N/A	18–24 months
Allendorf et al., (2018) [26]	Retrospective matched-control study	Bayley score-II, PDI and MDI	Medium	37	24	1039	Medical NEC 28 Surgical NEC 27	24 months
Muto et al., (2021) [21]	Retrospective study	K test	Medium	978	31	649.8	24.5	1.5 years corrected age / 3 years chronological age
Mondal et al., (2021) [17]	Retrospective study	Cognitive impairment, motor impairment, speaking difficulties, cerebral palsy, behavioural, visual impairment, seizures	Low	18	15	N/A	N/A	11.2 years

*MDI* Mental Developmental Index, *PDI* Psychomotor Developmental Index, *IQ* Intelligence Quotient, *DAS-II* Differential Ability Scales II, *LPA* Latent Profile Analysis, *WISC-III-NL* Wechsler Intelligence Scale for Children, *M-ABC* Movement Assessment Battery for Children, *IVH* Intraventricular Haemorrhage, *PVL* Periventricular Leukaemia, *BSID-II K*-*ASQ*, Korean Ages and Stages Questionnaire; *K-DST*, Korean Developmental Screening Test, *ASQ*, Ages and Stage Analysis; *K test*, Kyoto Scale of Physiological Development

### Quality of included studies

The majority of papers had only low or medium risk of bias. Two studies (Cuzilla et al., 2014, Ganapathy et al., 2013) were assessed to be of high risk of bias due to incomplete studies and follow-up [11, 12].

Included studies reported a variety of measures of neurodevelopmental outcome. These included formal developmental assessments, including the Bayley score, the diagnosis of a neurodisability (such as cerebral palsy) or surrogate markers, such as the presence or absence of white matter brain injury (WMBI) on imaging. Where comparisons were made, infants with NEC undergoing surgical intervention consistently had an increased risk of neurodevelopmental impairment compared to those treated without surgery [5, 8, 9, 11, 13]. Further, infants undergoing surgery had higher rates of intraventricular haemorrhage (IVH) and periventricular leukomalacia (PVL) compared to medically managed groups [8, 14–20].

Several pre-operative factors were universally associated with neurodevelopmental outcomes. These included non-modifiable factors related to the babies’ underlying condition or status, such as: gestational age at birth, birth weight, and age of NEC onset (Table 3). Extreme prematurity (< 28 week gestation at birth) and lower birth weight were associated with worse neurodevelopmental outcomes [6, 8, 13, 30]. Two studies reported that an earlier age of NEC onset from birthdate was significantly associated with subsequent development of WMBI [6, 9].

**Table 3:** Non-modifiable and modifiable factors identified

Non-modifiable	References	Modifiable	References
Gestational age: Lower gestational age at birth is associated with worse neurodevelopmental outcomes	Martin et al. (2010) [30] , Roze et al. (2011) [20] , Shah et al. (2011), Wadhawan et al. (2013) [14], Cuzzilla et al. (2014), [11] Merhar et al. (2014), [9] Fullerton et al. (2018) ,[8] Humberg et al. (2020), [15] Blakely et al. (2021), [28] Mondal et al. (2021),[17] Muto et al. (2021) [21]	Postnatal steroid use: Steroid use was associated with higher grades of WMBI	Garg et al. (2021) [6]
Birthweight: Lower birth weight is associated with worse neurodevelopmental outcomes	Hintz et al. (2005) [13] Roze et al. (2011) [20] Shah et al. (2011) Wadhawan et al. (2013) [14] Fullerton et al. (2018) [8] Humberg et al. (2020) [15] Garg et al. (2021) [6]	Prophylactic miconazole use: Miconazole use was associated with a lower intestinal perforation rate and improved neurodevelopmental outcomes	Muto et al. (2021) [21]
Age of NEC onset: Earlier onset of NEC is associated with worse neurodevelopmental outcomes	Merhar (2014) [9] Garg et al. (2021) [6]	Red cell transfusion: Transfusion prior to NEC onset was associated with worse neurodevelopmental outcomes	Garg et al. (2021) [6] Muto et al. (2021) [21]
Race: Infants from a black ethnic group were more likely to undergo surgical intervention	Shah TA (2011) [18]	Post-operative skin-to-skin contact: Lack of skin-to-skin contact was associated with worse outcomes	Garg et al. (2021) [6] Muto et al. (2021) [21]
		Peritoneal drain pre- or post-laparotomy: The use of a drain was associated with higher grades of WMBI	Garg et al. (2021) [6]
		Surgical procedures: Enterostomy formation was associated with worse neurodevelopmental outcomes whereas primary anastomosis with better outcomes	Garg et al. (2021) [6] Muto et al. (2021) [21] Roze et al. (2011) [20]

A recurring finding was that the majority of neonates undergoing laparotomy did not subsequently reach age-appropriate milestones, and potentially modifiable factors in the surgical management of NEC were reported to be associated with this [6, 21]. The use of an intra-peritoneal drain pre- or post-laparotomy was associated with increased WBMI in an sNEC cohort [6]. Following bowel resection, infants undergoing enterostomy formation had significantly worse neurodevelopmental outcomes when compared to infants undergoing primary anastomosis reported as both lower IQ score and a higher incidence of WMBI [20].

Potentially modifiable but not directly surgical factors in the management of babies with sNEC included the use of pharmacological agents. The postnatal use of steroids was associated with higher incidence of WMBI and the prophylactic use of miconazole was associated with a reduction in the incidence of bowel perforation in NEC infants, subsequently reducing the need for surgical intervention [6, 21]. Other factors reported to be associated with worse neurodevelopmental outcomes included red cell transfusion and a lack of skin-to-skin contact post-operatively [6, 21] (Table 3).

### Data aggregation

Due to the heterogeneity of both the patients and neurodevelopmental outcomes reported in the studies included in the review, no data aggregation or further analysis was possible.

## Discussion

We present a systematic review of the current evidence for associations between both patient and treatment-related factors and neurodevelopmental outcomes in infants with sNEC. In keeping with previous work, this confirms worse neurodevelopmental outcomes for infants undergoing surgery for NEC than those managed without surgery. The review also identifies a number of aspects of babies’ care that relate to later neurodevelopmental outcomes. Several are patient related and not amenable to modification such as lower gestational age and birth weight. Others do offer potential for change though, including the use of drains and enterostomy.

Whilst all studies in our review included data on modifiable and non-modifiable factors, few analysed their association with neurodevelopmental outcomes specific to surgical cohorts. In addition, the 22 studies included in the review reported very heterogeneous results regarding both patient characteristics and details of treatment delivered, making comparisons challenging.

Considering non-modifiable risk factors, it is unsurprising to see universally worse neurodevelopmental outcomes in association with lower gestational age and birth weight. A smaller and less mature baby will have less reserve, and hence greater vulnerability to the effects of NEC. As we continue to see more babies of extremely low gestational age undergo surgery, it seems likely that we will see more long-term neurodevelopmental morbidity in survivors [32]. This will have health service planning implications as these babies grow into childhood and beyond.

The surgical strategies for NEC that are reported as being associated with worse neurodevelopmental outcomes (the use of drains and enterostomies rather than primary anastomosis) potentially offer an opportunity to change practice to improve outcomes [6, 20]. The underlying state of the baby may well be a significant confounding factor in both these examples, with drains being reserved for the sickest babies and primary anastomosis only being possible in a more stable and robust baby. Nevertheless, enterostomies can lead to more challenging fluid and electrolyte management in neonates, and it is known that their presence in neonates is associated with poor somatic growth during a crucial phase in brain development, especially in the presence of inflammation [33, 34]. It is therefore plausible that avoiding an enterostomy could help protect brain growth and improve neurodevelopmental outcomes. Alternative surgical strategies such as damage limitation and second look laparotomies that avoid enterostomies may have potential to lead to better neurodevelopmental outcomes [35].

Non-surgical treatment factors reported to be associated with worse outcomes are very likely to be confounded by the characteristics of the babies in question. For example, postnatal steroids are associated with worse outcomes but are reserved for the sickest babies [6]. It cannot be concluded that steroids should be avoided to improve neurodevelopmental outcomes in sNEC. Likewise, the association of worse outcomes with blood transfusion and an inability to have skin-to-skin contact post-operatively almost certainly reflects the condition of the babies rather than a direct causal effect [6, 21].

The chief strength of our review lies in its size. Using broad search terms, we identified a large number of papers for screening and so can be confident that we have included all relevant articles. The articles included in the review present data on a total of 49,426 babies from a wide geographical spread, making it likely that conclusions will be broadly applicable. However, only 6 of the 22 papers included used prospectively collected data, and the remainder were retrospective, with the exception of 1 prognosis study. The review is further hampered by the heterogeneity of the studies included, both in the factors that may affect outcome that they included and in the outcome tools or measures that they use. Several did not use a formal neurodevelopment assessment tool and those that did used a variety of different ones. This has precluded aggregation of the data into a meta-analysis. The follow-up in some studies is also rather limited, and only 4 of the 22 studies followed children into their school age years, where the impact of neurodevelopmental issues is more fully exhibited [15, 18, 24, 29]. Bias, however, was not a major concern, with only two included papers being at high risk of bias due to inadequate follow-up.

Further research is needed to determine modifiable factors that influence neurodevelopmental outcome in sNEC cohorts. Ideally, these studies should explore the influence of factors, including blood transfusion, steroid, and antifungal use. In addition, a large-scale, multicentre, prospective study investigating the impact of different surgical strategies on neurodevelopmental outcomes in NEC infants would be of great interest to surgeons. These will need to include detailed data collection on the characteristics of the babies treated as well as the interventions carried out to be able to identify confounding variables. Follow-up of at least 2 years is required to adequately show neurodevelopmental impacts of NEC, but ideally 5 year follow-up would be completed to give a picture of longer term neurodevelopment. The use of standardised outcomes in future studies would greatly assist the comprehension and assessment of reported findings. Although not designed specifically for use in babies undergoing surgery, and indeed including the development of NEC as an outcome in itself, the Core Outcomes in Neonatology outcome set published in 2020 is a helpful tool for researchers [36]. It provides a range of relevant and measurable outcomes including longer term neurodevelopmental outcomes, and whilst it may be that future work specifically on surgical intervention in NEC requires modifications or additions to this twelve point, it should form the foundation of any future work in the area.

## Supplementary Information

Below is the link to the electronic supplementary material.

Supplementary file1 (DOCX 14 KB)

Supplementary file2 (DOCX 17 KB)

## References

[1] Duchon J, Barbian ME, Denning PW (2021) Necrotizing Enterocolitis. Clin Perinatol 48(2):229–250. 10.1016/j.clp.2021.03.002 34030811 10.1016/j.clp.2021.03.002

[2] Flahive C, Schlegel A, Mezoff EA (2020) Necrotizing enterocolitis: updates on morbidity and mortality outcomes. J Pediatr 220:7–9. 10.1016/j.jpeds.2019.12.035 31955884 10.1016/j.jpeds.2019.12.035

[3] Allin B, Long AM, Gupta A et al (2019) A UK wide cohort study describing management and outcomes for infants with surgical necrotising enterocolitis. Sci Rep. 10.1038/srep41149 28128283 10.1038/srep41149 PMC5269581

[4] Jones I, Hall N (2020) Contemporary outcomes for infants with necrotising enterocolitis - a systematic review. J Pediatr. 10.1016/j.jpeds.2019.11.011 31982088 10.1016/j.jpeds.2019.11.011

[5] Rees CM, Pierro A, Eaton S (2007) Neurodevelopmental outcomes of neonates with medically and surgically treated necrotizing enterocolitis. Arch Dis Child 92(3):193–198. 10.1136/adc.2006.099929 16984980 10.1136/adc.2006.099929 PMC2675329

[6] Garg PM, Paschal JL, Zhang M et al (2021) Brain injury in preterm infants with surgical necrotizing enterocolitis: clinical and bowel pathological correlates. Pediatr Res 91(5):1182–1195. 10.1038/s41390-021-01614-3 34103675 10.1038/s41390-021-01614-3 PMC10308193

[7] Robinson JR, Rellinger EJ, Hatch LD et al (2017) Surgical necrotizing enterocolitis. Semin Perinatol 41(1):70–79. 10.1053/j.semperi.2016.09.020 27836422 10.1053/j.semperi.2016.09.020 PMC5777619

[8] Fullerton BS, Hong CR, Velazco CS et al (2018) Severe neurodevelopmental disability and healthcare needs among survivors of medical and surgical necrotizing enterocolitis: a prospective cohort study. J Pediatr Surg S0022–3468(17):30651–30656. 10.1016/j.jpedsurg.2017.10.029 10.1016/j.jpedsurg.2017.10.029 29079317

[9] Merhar SL, Ramos Y, Meinzen-Derr J et al (2014) Brain magnetic resonance imaging in infants with surgical necrotizing enterocolitis or spontaneous intestinal perforation versus medical necrotizing enterocolitis. J Pediatr 164(2):410–412. 10.1016/j.jpeds.2013.09.055 24210927 10.1016/j.jpeds.2013.09.055

[10] Grooten A, Tseli E, Äng O et al (2019) Elaborating on the assessment of the risk of bias in prognostic studies in pain rehabilitations using QUIPS–aspects of interrater agreement. BioMed Central. 10.1186/s41512-019-0050-0 10.1186/s41512-019-0050-0 PMC6460536 31093575

[11] Cuzzilla R, Dinsdale E, Moore A (2014). Neurodevelopmental outcomes of very low birth weight infants with necrotising enterocolitis; a comparison of surgical management with peritoneal drain or initial laparotomy. Journal of Paediatrics and Child Health. http://ovidsp.ovid.com/ovidweb.cgi?T=JS&PAGE=reference&D=emed15&NEWS=N&AN=71598138.

[12] Ganapathy V, Hay JW, Kim JH et al (2013) Long term healthcare costs of infants who survived neonatal necrotizing enterocolitis: a retrospective longitudinal study among infants enrolled in texas medicaid. BMC Pediatr 13:127. 10.1186/1471-2431-13-127 23962093 10.1186/1471-2431-13-127 PMC3765805

[13] Hintz SR, Kendrick DE, Stoll BJ et al (2005) Neurodevelopmental and growth outcomes of extremely low birth weight infants after necrotizing enterocolitis. Paediatrics 115(3):696–703. 10.1542/peds.2004-0569 10.1542/peds.2004-0569 15741374

[14] Wadhawan R, Oh W, Hintz SR et al (2013) Neurodevelopmental outcomes of extremely low birth weight infants with spontaneous intestinal perforation or surgical necrotizing enterocolitis. J Perinatol 34(1):64–70. 10.1038/jp.2013.128 24135709 10.1038/jp.2013.128 PMC3877158

[15] Humberg A, Spiegler J, Fortmann MI et al (2020) Surgical necrotizing enterocolitis but not spontaneous intestinal perforation is associated with adverse neurological outcome at school age. Sci Rep. 10.1038/s41598-020-58761-6 32047169 10.1038/s41598-020-58761-6 PMC7012917

[16] Adesanya OA, O’Shea TM, Turner CS et al (2005) Intestinal perforation in very low birth weight infants: growth and neurodevelopment at 1 year of age. J Perinatol 25(9):583–589. 10.1038/sj.jp.7211360 16034475 10.1038/sj.jp.7211360

[17] Mondal A, Misra D, Al-Jabir A et al (2021). Necrotizing enterocolitis in neonates: Has the brain taken a hit 10 years later? Journal of Pediatric Neurosciences 16(1):30–34. https://www.pediatricneurosciences.com/article.asp?issn=1817-1745;year=2021;volume=16;issue=1;spage=30;epage=34;aulast=Mondal. 10.4103/jpn.JPN_41_20 PMC8276955 34316305

[18] Shah TA, Meinzen-Derr J, Gratton T et al (2011) Hospital and neurodevelopmental outcomes of extremely low-birth-weight infants with necrotizing enterocolitis and spontaneous intestinal perforation. J Perinatol 32(7):552–558. 10.1038/jp.2011.176 22157625 10.1038/jp.2011.176 PMC3496418

[19] Arnold M, Moore SW, Sidler D et al (2010) Long-term outcome of surgically managed necrotizing enterocolitis in a developing country. Pediatr Surg Int 26(4):355–360. 10.1007/s00383-010-2583-8 20204650 10.1007/s00383-010-2583-8

[20] Roze E, Ta BDP, van der Ree MH et al (2011) Functional impairments at school age of children with necrotizing enterocolitis or spontaneous intestinal perforation. Pediatr Res 70(6):619–625. 10.1203/PDR.0b013e31823279b1 21857378 10.1203/PDR.0b013e31823279b1

[21] Muto M, Sugita K, Ibara S et al (2021) Discrepancy between the survival rate and neurophysiological development in postsurgical extremely low-bith-weight infants: a retrospective study over two decades at a single institution. Pediatr Surg Int 37:411–417. 10.1007/s00383-020-04825-7 33427921 10.1007/s00383-020-04825-7

[22] Niemarkt HJ, De Meij TG, van Ganzewinkel C et al (2019) Necrotizing enterocolitis, gut microbiota, and brain development: role of the brain-gut axis. Neonatology 115(4):423–431. 10.1159/000497420 30974443 10.1159/000497420 PMC6604259

[23] Keunen K, Sperna Weiland NH, Bakker BS et al (2022) Impact of surgery and anesthesia during early brain development: a perfect storm. Pediatr Anesth 32(6):697–705. 10.1111/pan.14433 10.1111/pan.14433 PMC9311405 35266610

[24] Vaidya R, Jensen E, Joseph RM et al (2022) Long term Outcome of Necrotising enterocolitis and spontaneous intestinal perforation. Pediatrics. 10.1542/peds.2022-056445 36200375 10.1542/peds.2022-056445 PMC9647591

[25] Bos AF (2013) Bayley-II or Bayley-III: what do the scores tell us? Dev Med Child Neurol 55(11):978–979. 10.1111/dmcn.12234 23930736 10.1111/dmcn.12234

[26] Allendorf A, Dewitz R, Weber J et al (2018) Necrotizing enterocolitis as a prognostic factor for the neurodevelopmental outcome of preterm infants–match control study after 2 years. J Pediatr Surg 53(8):1573–1577. 10.1016/j.jpedsurg.2018.01.006 29409620 10.1016/j.jpedsurg.2018.01.006

[27] Ayed M, Shah P, Lodha A et al (2014) Outcome of Infants with necrotising enterocolitis (nec): the impact of laparotomy versus peritoneal drainage. Paediatr Child Health 19(6):51. 10.1093/pch/19.6.e35-42

[28] Blakely ML, Tyson JE, Lally KP et al (2006) Laparotomy versus peritoneal drainage for necrotizing enterocolitis or isolated intestinal perforation in extremely low birth weight infants: outcomes through 18 months adjusted age. Pediatrics 117(4):680–687. 10.1542/peds.2005-1273 10.1542/peds.2005-1273 16549503

[29] Han SM, Knell J, Henry O et al (2020) Long-term outcomes of severe surgical necrotizing enterocolitis. J Pediatr Surg 55(5):848–851. 10.1016/j.jpedsurg.2020.01.019 32085915 10.1016/j.jpedsurg.2020.01.019

[30] Martin CR, Dammann O, Allred EN et al (2010) Neurodevelopment of extremely preterm infants who had necrotizing enterocolitis with or without late bacteremia. J pediatr 157(5):751–756. 10.1016/j.jpeds.2010.05.042 20598317 10.1016/j.jpeds.2010.05.042 PMC2952050

[31] shin sh, kim ek, kim sh et al (2021) Head growth and neurodevelopment of preterm infants with surgical necrotizing enterocolitis and spontaneous intestinal perforation. Children 8(10):833. 10.3390/children8100833 34682098 10.3390/children8100833 PMC8534747

[32] Vallant N, Haffenden V, Peatman O et al (2022) Outcomes for necrotising enterocolitis (NEC) in babies born at the threshold of viability: a case–control study. BMJ Paediatrics Open. 10.1136/bmjpo-2022-001583 36645754 10.1136/bmjpo-2022-001583 PMC9717317

[33] Chong C, van Druten J, Briars G et al (2019) Neonates living with enterostomy following necrotising enterocolitis are at high risk of becoming severely underweight. Eur J Paediatrics 178(12):1875–1881. 10.1007/s00431-019-03440-6 10.1007/s00431-019-03440-6 PMC6892362 31522315

[34] Davidson JR, Omran K, Chong CKL, Eaton S, Edwards AD, Yardley IE (2023) Exploring growth failure in neonates with enterostomy. J Pediatric Surg. 10.1016/j.jpedsurg.2023.10.010 10.1016/j.jpedsurg.2023.10.010 37940463

[35] Arul GS, Singh M, Ali AM et al (2019) Damage control surgery in neonates: lessons learned from the battlefield. J Paediatr Surg 54(10):2069–2074. 10.1016/j.jpedsurg.2019.04.001 10.1016/j.jpedsurg.2019.04.001 31103271

[36] Webbe JWH, Duffy JMN, Afonso E, Al-Muzaffar I, Brunton G, Greenough A, Hall NJ, Knight M, Latour JM, Lee-Davey C, Marlow N, Noakes L, Nycyk J, Richard-Löndt A, Wills-Eve B, Modi N, Gale C (2019) Core outcomes in neonatology: development of a core outcome set for neonatal research. Arch Dis Child Fet Neonatal Edit 105(4):425–431. 10.1136/archdischild-2019-317501 10.1136/archdischild-2019-317501 PMC7363790 31732683

