# Supplementary material for: Factors affecting neurodevelopmental outcome following surgical necrotising enterocolitis: a systematic review
Source: Pediatr Surg Int. 2024 Mar 6;40(1):71. doi: 10.1007/s00383-024-05651-x (PMC10917837; doi:10.1007/s00383-024-05651-x)
Supplement: Supplementary file 1 — Supplementary file1 (DOCX 14 KB) [file 383_2024_5651_MOESM1_ESM.docx]

Factors affecting neurodevelopmental outcome following surgical necrotising enterocolitis; a systematic review. Pediatric Surgery International. Okten E I, Frankl M, Wu S, Gamaty H, Thompson H, Yardley I E; King’s College London, GKT School of Medical Education, [miri.frankl@hotmail.co.uk](mailto:miri.frankl@hotmail.co.uk)

###### Appendix 1: Search strategy:

| Databases searched | Search Terms | Limits |
| --- | --- | --- |
| Embase | (Infant* or newborn* or new-born* or baby or babies* or preterm* or prematur* or pre-term (Abstract)) AND (necroti?ing enterocolitis OR NEC OR nec(Title)) AND (Surg* or operat* or resect* or laparotom* or peritoneal drain* or enterostom* (Abstract))  AND  (Neuro* or impair* or cogniti* (Abstract)) | - English language - Human studies - Studies 2000-current |
| MEDLINE | (Infant* or newborn* or new-born* or baby or babies* or preterm* or prematur* or pre-term (Abstract)) AND (necroti?ing enterocolitis OR NEC OR nec(Title)) AND (Surg* or operat* or resect* or laparotom* or peritoneal drain* or enterostom* (Abstract))  AND  (Neuro* or impair* or cogniti* (Abstract)) | - English language - Human studies - Studies 2000-current |
| Pubmed | (Infant* or newborn* or new-born* or baby or babies* or preterm* or prematur* or pre-term (Abstract)) AND (necroti?ing enterocolitis OR NEC OR nec(Title)) AND (Surg* or operat* or resect* or laparotom* or peritoneal drain* or enterostom* (Abstract))  AND  (Neuro* or impair* or cogniti* (Abstract))  (((("Cognition Disorders"[Mesh]) OR "Cognition"[Mesh]) OR "Neurology"[Mesh]) AND "Surgical Procedures, Operative"[Mesh]) AND "Enterocolitis, Necrotizing"[Mesh] | - English language - Human studies - Studies 2000-current |
