# Supplementary material for: Factors affecting neurodevelopmental outcome following surgical necrotising enterocolitis: a systematic review
Source: Pediatr Surg Int. 2024 Mar 6;40(1):71. doi: 10.1007/s00383-024-05651-x (PMC10917837; doi:10.1007/s00383-024-05651-x)
Supplement: Supplementary file 2 — Supplementary file2 (DOCX 17 KB) [file 383_2024_5651_MOESM2_ESM.docx]

Factors affecting neurodevelopmental outcome following surgical necrotising enterocolitis; a systematic review. Pediatric Surgery International. Okten E I, Frankl M, Wu S, Gamaty H, Thompson H, Yardley I E; King’s College London, GKT School of Medical Education, [miri.frankl@hotmail.co.uk](mailto:miri.frankl@hotmail.co.uk)

**References:**

[1] Duchon J, Barbian ME, Denning PW (2021). Necrotizing Enterocolitis. Clinical in Perinatology 48(2):229-250. https://doi.org/10.1016/j.clp.2021.03.002

[2] Flahive C, Schlegel A, Mezoff EA (2020).Necrotizing Enterocolitis: Updates on Morbidity and Mortality Outcomes. The Journal of Pediatrics 220:7–9. https://doi.org/10.1016/j.jpeds.2019.12.035

[3] Allin B, Long AM, Gupta A et al (2019). A UK wide cohort study describing management and outcomes for infants with surgical Necrotising Enterocolitis. Scientific Reports. https://doi.org/10.1038/srep41149.

[4] Jones, I and Hall N (2020). Contemporary Outcomes for Infants with Necrotising Enterocolitis - A Systematic Review. The Journal of Pediatrics. https://doi.org/10.1016/j.jpeds.2019.11.011

[5] Rees CM, Pierro A, Eaton S (2007). Neurodevelopmental outcomes of neonates with medically and surgically treated necrotizing enterocolitis. Archives of Disease in Childhood 92(3):193–198. doi: 10.1136/adc.2006.099929

[6] Garg PM, Paschal JL, Zhang M et al (2021). Brain injury in preterm infants with surgical necrotizing enterocolitis: clinical and bowel pathological correlates. Pediatric Research 91(5):1182–1195. <https://doi.org/10.1038/s41390-021-01614-3>

[7] Robinson JR, Rellinger EJ, Hatch LD et al (2017). Surgical necrotizing enterocolitis. Seminars in Perinatology 41(1):70–9. <https://doi.org/10.1053/j.semperi.2016.09.020>

[8] Fullerton BS, Hong CR, Velazco CS et al (2018). Severe neurodevelopmental disability and healthcare needs among survivors of medical and surgical necrotizing enterocolitis: A prospective cohort study. Journal of Pediatric Surgery S0022-3468(17):30651-30656. <https://doi.org/10.1016/j.jpedsurg.2017.10.029>

[9] Merhar SL, Ramos Y, Meinzen-Derr J et al (2014). Brain Magnetic Resonance Imaging in Infants with Surgical Necrotizing Enterocolitis or Spontaneous Intestinal Perforation versus Medical Necrotizing Enterocolitis. The Journal of Pediatrics 164(2):410-412. <https://doi.org/10.1016/j.jpeds.2013.09.055>

[10] Grooten A, Tseli E, Äng O et al (2019). Elaborating on the assessment of the risk of bias in prognostic studies in pain rehabilitations using QUIPS - aspects of interrater agreement. BioMed Central. https://doi.org/10.1186/s41512-019-0050-0.

[11] Cuzzilla R, Dinsdale E, Moore A (2014). Neurodevelopmental outcomes of very low birth weight infants with necrotising enterocolitis; a comparison of surgical management with peritoneal drain or initial laparotomy. Journal of Paediatrics and Child Health. <http://ovidsp.ovid.com/ovidweb.cgi?T=JS&PAGE=reference&D=emed15&NEWS=N&AN=71598138>.

[12] Ganapathy V, Hay JW, Kim JH et al (2013). Long term healthcare costs of infants who survived neonatal necrotizing enterocolitis: a retrospective longitudinal study among infants enrolled in Texas Medicaid. BMC Pediatrics 13:127. https://doi.org/10.1186/1471-2431-13-127

[13] Hintz SR, Kendrick DE, Stoll BJ et al (2005). Neurodevelopmental and Growth Outcomes of Extremely Low Birth Weight Infants After Necrotizing Enterocolitis. Paediatrics 115(3):696-703. https://doi.org/10.1542/peds.2004-0569

[14] Wadhawan R, Oh W, Hintz SR et al (2013). Neurodevelopmental outcomes of extremely low birth weight infants with spontaneous intestinal perforation or surgical necrotizing enterocolitis. Journal of Perinatology 34(1):64–70. https://doi.org/10.1038/jp.2013.128

[15] Humberg A, Spiegler J, Fortmann MI et al (2020). Surgical necrotizing enterocolitis but not spontaneous intestinal perforation is associated with adverse neurological outcome at school age. Scientific Reports. https://doi.org/10.1038/s41598-020-58761-6.

[16] Adesanya OA, O’Shea TM, Turner CS et al (2005). Intestinal Perforation in Very Low Birth Weight Infants: Growth and Neurodevelopment at 1 Year of Age. Journal of Perinatology 25(9):583–589. https://doi.org/10.1038/sj.jp.7211360

[17] Mondal A, Misra D, Al-Jabir A et al (2021). Necrotizing enterocolitis in neonates: Has the brain taken a hit 10 years later? Journal of Pediatric Neurosciences 16(1):30-34. https://www.pediatricneurosciences.com/article.asp?issn=1817-1745;year=2021;volume=16;issue=1;spage=30;epage=34;aulast=Mondal.

[18] Shah TA, Meinzen-Derr J, Gratton T et al (2011). Hospital and neurodevelopmental outcomes of extremely low-birth-weight infants with necrotizing enterocolitis and spontaneous intestinal perforation. Journal of Perinatology 32(7):552–558. https://doi.org/10.1038/jp.2011.176

[19] Arnold M, Moore SW, Sidler D et al (2010). Long-term outcome of surgically managed necrotizing enterocolitis in a developing country. Pediatric Surgery International 26(4):355–360. https://doi.org/10.1007/s00383-010-2583-8

[20] Roze E, Ta BDP, van der Ree MH et al (2011). Functional Impairments at School Age of Children With Necrotizing Enterocolitis or Spontaneous Intestinal Perforation. Pediatric Research 70(6):619–625. https://doi.org/10.1203/PDR.0b013e31823279b1

[21] Muto M, Sugita K, Ibara S et al (2021). Discrepancy between the survival rate and neurophysiological development in postsurgical extremely low-bith-weight infants: a retrospective study over two decades at a single institution. Pediatric Surgery International 37: 411-417. https://doi.org/10.1007/s00383-020-04825-7

[22] Niemarkt HJ, De Meij TG, van Ganzewinkel C et al (2019). Necrotizing Enterocolitis, Gut Microbiota, and Brain Development: Role of the Brain-Gut Axis. Neonatology 115(4):423–431. https://doi.org/10.1159/000497420.

[23] Keunen K, Sperna Weiland NH, Bakker BS et al (2022). Impact of surgery and anesthesia during early brain development: A perfect storm. Pediatric Anesthesia 32(6):697–705. https://doi.org/10.1111/pan.14433

[24] Vaidya R, Jensen E, Joseph RM et al (2022). Long term Outcome of Necrotising enterocolitis and spontaneous intestinal perforation. Pediatrics 150(5). https://doi.org/10.1542/peds.2022-056445

[25] Bos AF (2013). Bayley-II or Bayley-III: what do the scores tell us? Developmental Medicine and Child Neurology 55(11):978-979. <https://doi.org/10.1111/dmcn.12234>.

[26] Allendorf A, Dewitz R, Weber J et al (2018). Necrotizing enterocolitis as a prognostic factor for the neurodevelopmental outcome of preterm infants - match control study after 2 years. Journal of Pediatric Surgery 53(8):1573–1577. <https://doi.org/10.1016/j.jpedsurg.2018.01.006>

[27] Ayed M, Shah P, Lodha A et al (2014). Outcome of Infants with Necrotising Enterocolitis (NEC): The Impact of Laparotomy Versus Peritoneal Drainage. Paediatrics & Child Health 19(6):51. https://doi.org/10.1093/pch/19.6.e35-42.

[28] Blakely ML, Tyson JE, Lally KP et al (2006). Laparotomy Versus Peritoneal Drainage for Necrotizing Enterocolitis or Isolated Intestinal Perforation in Extremely Low Birth Weight Infants: Outcomes Through 18 Months Adjusted Age. PEDIATRICS 117(4):680–687. https://doi.org/10.1542/peds.2005-1273

[29] Han SM, Knell J, Henry O et al (2020). Long-term outcomes of severe surgical necrotizing enterocolitis. Journal of Pediatric Surgery 55(5):848–851. <https://doi.org/10.1016/j.jpedsurg.2020.01.019>

[30] Martin CR, Dammann O, Allred EN et al (2010). Neurodevelopment of Extremely Preterm Infants who had Necrotizing Enterocolitis with or without Late Bacteremia. The Journal of Pediatrics 157(5):751-756. <https://doi.org/10.1016/j.jpeds.2010.05.042>.

[31] Shin SH, Kim EK, Kim SH et al (2021). Head Growth and Neurodevelopment of Preterm Infants with Surgical Necrotizing Enterocolitis and Spontaneous Intestinal Perforation. Children 8(10):833. https://doi.org/10.3390/children8100833.

[32] Vallant N, Haffenden V, Peatman O et al (2022). Outcomes for necrotising enterocolitis (NEC) in babies born at the threshold of viability: a case–control study. BMJ Paediatrics Open 6(1). [https://doi.org/](https://doi.org/10.1016/j.jpeds.2010.05.042)10.1136/bmjpo-2022-001583.

[33] Chong C, van Druten J, Briars G et al (2019). Neonates living with enterostomy following necrotising enterocolitis are at high risk of becoming severely underweight. European Journal of Paediatrics 178(12):1875–1881. <https://doi.org/10.1007/s00431-019-03440-6>

[34] Davidson, J.R., Omran, K., Chong, C.K.L., Eaton, S., Edwards, A.D. and Yardley, I.E. (2023). Exploring Growth Failure in Neonates With Enterostomy. *Journal of Pediatric Surgery*, [online] pp.S0022-3468(23)006206. doi:https://doi.org/10.1016/j.jpedsurg.2023.10.010.

[35] Arul GS, Singh M, Ali AM et al (2019). Damage control surgery in neonates: Lessons learned from the battlefield. Journal of Paediatric Surgery 54(10):2069-2074. <https://doi.org/10.1016/j.jpedsurg.2019.04.001>

[36] Webbe, J.W.H., Duffy, J.M.N., Afonso, E., Al-Muzaffar, I., Brunton, G., Greenough, A., Hall, N.J., Knight, M., Latour, J.M., Lee-Davey, C., Marlow, N., Noakes, L., Nycyk, J., Richard-Löndt, A., Wills-Eve, B., Modi, N. and Gale, C. (2019). Core outcomes in neonatology: development of a core outcome set for neonatal research. *Archives of Disease in Childhood - Fetal and Neonatal Edition*, 105(4), pp.425–431. doi:https://doi.org/10.1136/archdischild-2019-317501.
